# Social Media and Adolescent Mental Health: A Comprehensive Narrative Review

**DOI:** 10.7759/cureus.103089

**Published:** 2026-02-06

**Authors:** Vinod Sharma, Aditi Sharma

**Affiliations:** 1 Psychiatry, Geisinger Commonwealth School of Medicine, Scranton, USA; 2 Psychiatry, The Wright Center for Graduate Medical Education, Scranton, USA

**Keywords:** adolescents, anxiety, body image, depression, digital literacy, mental health, peer support, social media

## Abstract

Social media use is nearly universal among adolescents and has become a prominent focus of concern regarding its potential impact on mental health. The aim of this narrative review is to synthesize and critically evaluate the evidence on the relationship between social media use and adolescent mental health, with particular attention to risk pathways, protective factors, underlying mechanisms, moderating variables, and intervention strategies. Existing research suggests that social media may function as both a risk factor and a source of support, depending on patterns of use, individual vulnerability, and contextual influences. A narrative review of the literature was conducted using PubMed, Google Scholar, and PsycINFO to identify peer-reviewed articles published between 2007 and 2025. Search terms included social media, adolescents, mental health, depression, anxiety, sleep, cyberbullying, body image, problematic use, and digital interventions. Systematic reviews, meta-analyses, longitudinal studies, experimental trials, and high-quality observational studies were prioritized, and findings were synthesized thematically. Social media influences adolescent mental health through multiple risk and protective pathways, with modest individual-level effects but meaningful population-level relevance due to widespread use during a sensitive developmental period. Outcomes are shaped by psychological mechanisms, moderating factors, and patterns of engagement, while emerging multilevel interventions show promise in reducing harm and enhancing benefits. Overall, social media is not inherently harmful to adolescent mental health; rather, its impact depends on how, why, and in what context it is used, underscoring the need for nuanced, developmentally informed, and multilevel approaches.

## Introduction and background

Over the past two decades, adolescent social media engagement has shifted from a niche online activity into a central feature of everyday life. Adolescence, typically defined as ages 10-19 years, is characterized by pubertal and hormonal maturation, heightened self-consciousness, increased sensitivity to peer evaluation, and ongoing neurobiological development of reward-processing and emotion-regulation systems, factors that increase vulnerability to internalizing mental health conditions such as depression and anxiety [1-3]. Mental health refers to emotional, psychological, and social well-being that enables effective functioning, whereas mental health conditions reflect clinically significant disturbances associated with functional impairment [1]. Social media refers to digital platforms that allow users to create, share, and interact with user-generated content and social networks in real time [4]. During adolescence, these platforms represent developmentally salient social environments that intersect with identity formation, peer relationships, emotional regulation, and social comparison processes [5]. Epidemiologic data demonstrate a marked rise in mental health conditions across adolescence, with the lifetime prevalence of major depressive disorder increasing from approximately 5-8% in early adolescence to 15-20% by late adolescence, and anxiety disorders affecting nearly one in three adolescents by age 18 [3].

Access to social media has expanded rapidly with widespread smartphone adoption, replacing earlier reliance on shared computers or internet cafés. Adolescents now engage primarily through personal mobile devices, enabling near-continuous connectivity, real-time interaction, and increased privacy, which may heighten emotional investment and reduce adult oversight during a sensitive developmental period [6,7]. Most adolescents report daily use averaging 3-5 hours, with nationally representative U.S. data showing a sharp age-related increase in cumulative exposure, from approximately 5.5 hours/day among children aged 8-12 years to nearly 9 hours/day among adolescents aged 13-18 years [6,7]. Early disclosure on adolescent social media use was polarized, with concerns emphasizing excessive screen time defined as cumulative exposure to digital screens across social and recreational activities, and fears of a broader “digital mental health crisis,” describing rising rates of adolescent depression and anxiety alongside rapid technological expansion, though causal relationships remain debated [1,7]. In contrast, more optimistic perspectives highlighted social media’s potential to support social connection, identity exploration, and youth empowerment [1]. However, large-scale reviews indicate that associations between social media use and mental health outcomes are generally small but consistent, and depend more on patterns and contexts of use than total time spent online [7]. These developmental vulnerabilities intersect with digital environments intentionally designed to maximize engagement through personalized algorithms, intermittent reinforcement, rapid content delivery, and social feedback mechanisms such as likes and comments [8,9]. Because socioemotional and reward systems mature earlier than cognitive control, adolescents may be particularly susceptible to these features, amplifying emotional reactivity to online experiences [10,11]. Contemporary evidence therefore supports a dual-edged model: social media use is associated with internalizing symptoms, body-image disturbance, sleep disruption, cyberbullying exposure, and problematic or addictive use-especially among emotionally vulnerable youth [8,12,13], while also facilitating social connection, peer support, identity exploration, and access to mental health information, particularly for adolescents with limited offline support or marginalized identities [9,14]. Outcomes are moderated by individual characteristics, contextual factors, platform design, and cultural norms [1,7]. Cultural context further contributes to variability in mental health outcomes associated with social media use. Norms related to emotional expression, body image, peer relationships, and help-seeking differ across cultures and may influence how adolescents interpret and respond to online interactions. Appearance-based social comparison may exert stronger effects in cultural contexts emphasizing physical attractiveness or social status, whereas collectivist cultures may place greater salience on peer approval, conformity, and online group belonging. Cross-cultural research also suggests that parental monitoring practices, stigma surrounding mental health, and differential access to digital resources can moderate both risk and protective effects of social media use, contributing to heterogeneity in observed outcomes [1,7,11]. Despite a growing literature, a key research gap remains in synthesizing how active versus passive patterns of use differentially affect adolescent mental health beyond overall exposure time. Accordingly, this narrative review aims to synthesize evidence on risk and protective pathways, underlying psychological and technological mechanisms, and moderating factors, to inform psychiatric assessment, prevention, and intervention strategies.

## Review

Methods

A narrative review was conducted to synthesize evidence on the relationship between social media use and adolescent mental health. Electronic searches were performed using PubMed, Google Scholar, and PsycINFO for peer-reviewed articles published between January 2007 and March 2025. Search terms included combinations of social media, screen time, adolescents, mental health, depression, anxiety, sleep, cyberbullying, body image, problematic use, and digital interventions. Included articles consisted of systematic reviews, meta-analyses, longitudinal studies, randomized and quasi-experimental trials, and high-quality observational studies focusing on adolescents or young adults (approximately ages 10-25). Priority was given to studies reporting effect sizes, mechanistic insights, or clinical and public health relevance. Editorials, opinion pieces, non-peer-reviewed sources, and studies not available in English were excluded. Given the narrative (non-systematic) design, formal risk-of-bias assessment and PRISMA procedures were not applied. Although both narrative and literature reviews involve structured searches, narrative reviews emphasize interpretive synthesis and conceptual integration across heterogeneous evidence, whereas literature reviews are primarily descriptive summaries of existing studies. A narrative approach was therefore chosen to examine mechanisms, moderators, and clinical implications within a rapidly evolving field. Findings were synthesized thematically, focusing on risk pathways, protective factors, underlying mechanisms, moderating variables, and intervention evidence relevant to adolescent mental health.

Narrative review

Epidemiology of Adolescent Social Media Use

For the last 15 years, more than 90% of adolescents in North America and Europe have reported daily social media use, with a substantial proportion describing “almost constant” engagement [13]. This widespread adoption coincides with unprecedented access to mobile devices, algorithm-driven content, and persistent digital connectivity [1,6]. Social media engagement begins earlier than ever; children as young as 10 report active use of social platforms despite age restrictions, and by mid-adolescence, usage patterns increasingly resemble those of adults [6,12]. Platform preferences have shifted considerably across the 2007-2025 period, with early cohorts favoring platforms such as MySpace and Facebook and contemporary adolescents preferring visually oriented and short-form video platforms, including TikTok, Instagram, Snapchat, and YouTube [6,15]. These platforms emphasize rapid content refresh, emotionally engaging aesthetics, and salient social comparison cues such as likes, comments, filters, and follower counts, features central to user engagement and algorithmic amplification [10,15].

Importantly, mere time spent on social media is only weakly associated with mental health outcomes [12]. Instead, what adolescents do online and how they emotionally engage with digital interactions appear far more predictive of psychological well-being. Adolescents most commonly browse appearance-focused content (e.g., beauty, fitness, lifestyle influencers), peer-generated social content, short-form entertainment videos, and algorithmically curated feeds that prioritize emotionally salient material [6,13,15]. Exposure to idealized images and appearance-based feedback has been consistently linked to heightened social comparison, body dissatisfaction, and internalizing symptoms, particularly among girls [13,16]. Patterns such as passive consumption, nighttime engagement with emotionally arousing content, and exposure to peer conflict or cyberbullying are further associated with sleep disruption, mood disturbance, and increased risk for depression and anxiety [17]. Together, these findings underscore that content characteristics and engagement patterns, rather than overall duration of use, are central to understanding social media-related mental health risk in adolescents.

Instead, what adolescents do online, and how they feel about their digital interactions, proves far more predictive. Research consistently shows that specific patterns such as passive consumption, appearance-focused content, nighttime use, and exposure to cyberbullying are linked to poorer mental-health outcomes [12]. Conversely, active communication with friends, creative expression, and participation in supportive online communities correlate with enhanced well-being [16]. The omnipresence of social media has also normalized multitasking, constant notifications, and rapid shifts in attention, all of which may have downstream effects on executive functioning, emotional regulation, and sleep patterns. These epidemiological realities create both opportunity and vulnerability for today’s youth [8,9,17].

Risk Pathways

Evidence from narrative reviews, systematic reviews, and meta-analyses published between 2007 and 2025 consistently demonstrates that social media contributes to adolescent mental health risk through multiple, overlapping pathways. In their umbrella review of reviews, Odgers and Przybylski emphasized that while overall associations between screen time and mental health outcomes are modest, emotionally invested and maladaptive patterns of social media use show more reliable links to depression and anxiety than time-based measures alone [1,12]. Similarly, Verduyn et al. distinguished between passive and active use, demonstrating that passive consumption, such as scrolling through curated peer content without interaction, is more strongly associated with declines in subjective well-being and increases in negative affect [16]. At the individual symptom level, Vogel et al. showed that social comparison on social networking sites predicts lower self-esteem, which in turn mediates associations with depressive symptoms [13]. Adolescents engaging in reassurance seeking or ruminative posting may repeatedly expose themselves to social evaluation, reinforcing negative self-appraisals.

Body-image disturbance represents a particularly salient risk pathway following the rise of image-centric platforms. Verduyn et al. and Kross et al. highlighted that exposure to idealized and filtered images intensifies upward appearance-based social comparison, especially among girls and gender-diverse youth [15,16]. Experimental findings summarized in these reviews demonstrate that even brief exposure to idealized images can acutely worsen body satisfaction, underscoring the potency of this mechanism despite relatively short exposure durations. Sleep disruption has emerged as one of the most robust mediators linking social media use to mental health outcomes. Levenson et al. found that social media use before bedtime was associated with delayed sleep onset and poorer sleep quality, effects that were independent of total daily use [17]. Complementing this, Scott and Woods demonstrated that fear of missing out (FOMO) contributes to nighttime checking behaviors, which in turn predict sleep disturbance and next-day mood symptoms [18]. These findings align with earlier developmental sleep research by Short et al., which emphasizes the sensitivity of adolescent sleep architecture to behavioral and environmental disruption [5].

Cyberbullying and online harassment constitute another high-impact pathway with consistently larger effect sizes than general social media use [19]. Ophir et al. and Uncapher et al. demonstrated that repeated exposure to emotionally salient digital stressors impairs cognitive control and emotional regulation, mechanisms that may amplify the psychological impact of cyberbullying [8,9]. Reviews of epidemiological studies indicate that victims of cyberbullying experience substantially elevated rates of depression, anxiety, self-harm, and suicidal ideation, while perpetrators also exhibit increased emotional dysregulation and behavioral difficulties [8,9]. Finally, problematic or addictive social media use has been conceptualized as a distinct risk phenotype. Montag et al. identified platform features such as intermittent reinforcement, social reward cues, and infinite scroll as contributors to compulsive engagement patterns [10]. Meta-analytic evidence summarized in this work indicates moderate associations between problematic use and depression or anxiety, exceeding those observed for general use frequency and extending to academic impairment, loneliness, sleep disturbance, and reduced impulse control.

Protective and Supportive Pathways

**Figure 1:**
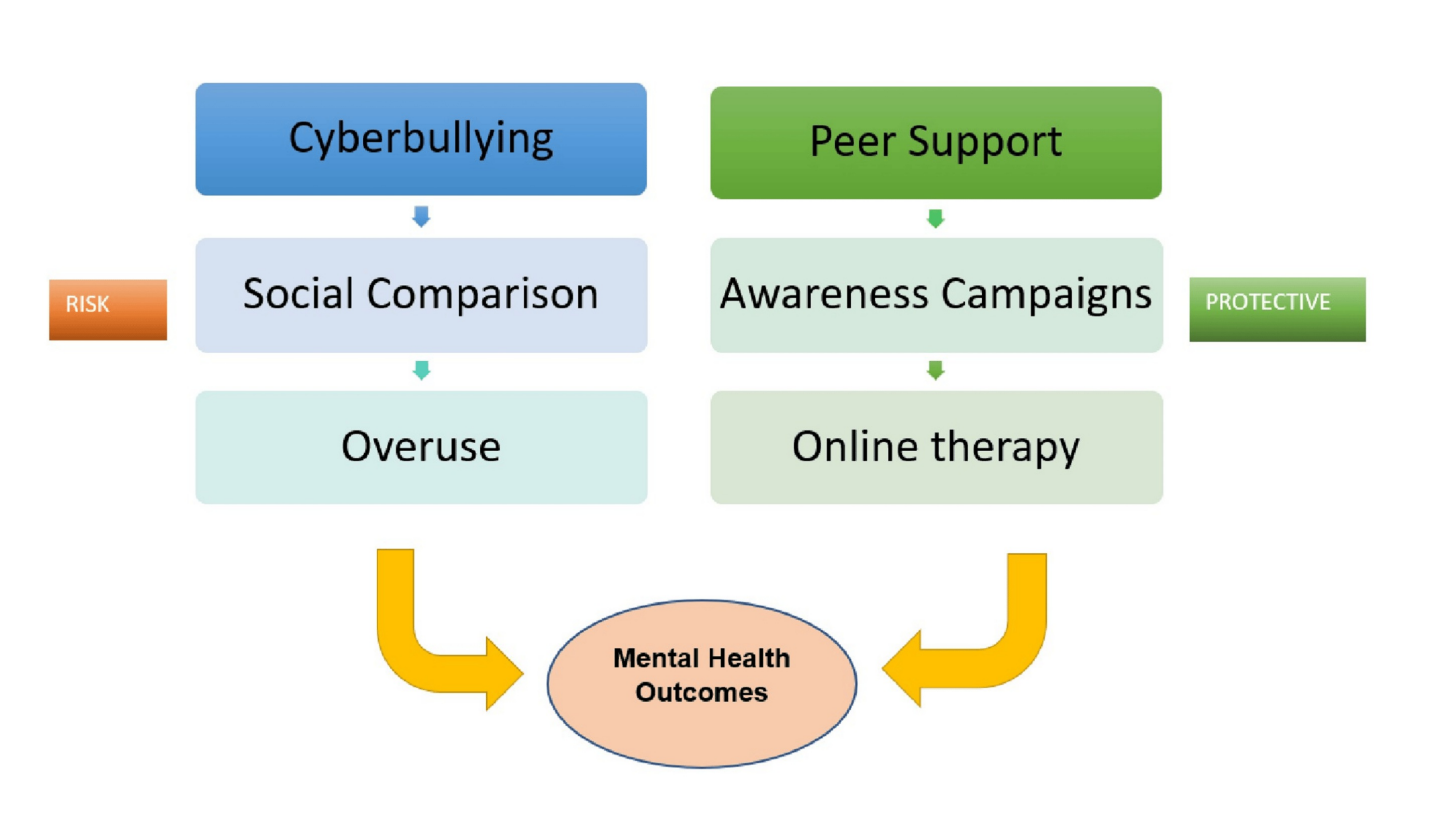
Risk and Protective Factors Influencing Adolescent Mental Health Outcomes This is an original figure created by the author using Microsoft Word.

Despite extensive focus on risk, a substantial body of literature highlights protective and resilience-promoting pathways associated with adolescent social media use. Large-scale longitudinal studies reviewed by George et al. indicate that digital media use can support well-being when it facilitates social connection rather than displacing offline relationships [11]. Adolescents frequently use social media to maintain friendships, coordinate social activity, and access emotional support, particularly outside of school hours. These effects appear most beneficial for adolescents with limited offline peer networks or social anxiety, consistent with a compensatory social hypothesis [5]. Identity exploration and affirmation represent another key protective pathway. Przybylski et al. and Baker et al. noted that online environments allow adolescents to explore emerging identities and values with greater autonomy and reduced immediate social risk [12,14]. For marginalized youth, including LGBTQ+ adolescents, online spaces may provide access to affirming communities unavailable in offline contexts, promoting identity coherence and psychological safety. Narrative and observational studies summarized by Kross et al. indicate that such affirming engagement is associated with reduced internalizing symptoms and increased perceived support [15]. Access to mental health information is an additional benefit increasingly documented in recent literature. Adolescents commonly encounter psychoeducational content, coping strategies, and peer narratives through social media. Kross et al. emphasized that exposure to credible mental health content can normalize help-seeking and reduce stigma, particularly when information is delivered through relatable peer or influencer narratives rather than traditional clinical messaging [15]. Emerging digital interventions, including mindfulness-based applications evaluated by Huberty et al., demonstrate small-to-moderate improvements in stress and emotion regulation, supporting the feasibility of leveraging digital platforms for prevention and early intervention [20]. These risk and protective pathways are illustrated in Figure 1.

Mechanisms Linking Social Media Use to Mental Health

Several interacting mechanisms explain social media’s dual-edged impact. Social comparison remains one of the most robustly supported mechanisms. Vogel et al. demonstrated that frequent comparison to idealized peer content undermines self-esteem, particularly in appearance- and popularity-focused contexts [13]. Verduyn et al. further clarified that passive consumption intensifies these effects, whereas active engagement may buffer them [16]. FOMO represents a closely related cognitive-emotional process. Przybylski et al. conceptualized FOMO as a stable motivational trait associated with anxiety and compulsive checking behaviors [12], while Baker et al. demonstrated its associations with depressive symptoms and somatic complaints [14]. These findings suggest that FOMO both motivates excessive use and mediates its negative psychological effects. From a pathophysiological perspective, these processes likely involve heightened reactivity of reward and stress-related neural systems, with impaired top-down regulation during adolescence contributing to vulnerability to mood and anxiety symptoms [14,16]. Technological design features further amplify these mechanisms. Kross et al. and Montag et al. described how algorithmic personalization, notifications, and reward-based feedback loops increase engagement intensity and emotional arousal, thereby increasing exposure to both supportive and harmful content [10,15]. Cognitive neuroscience research by Ophir et al. and Uncapher et al. indicates that frequent multitasking across digital platforms impairs sustained attention and working memory, potentially exacerbating academic stress and emotional dysregulation [8,9].

Moderators of Social Media Impact

The psychological impact of social media is not uniform but moderated by individual and contextual factors. Gender differences are consistently reported, with girls showing stronger associations between social media use and internalizing symptoms [21]. Vogel et al. and Verduyn et al. attributed this vulnerability partly to greater engagement in appearance-based comparison and sensitivity to peer feedback [13,16]. Developmentally, younger adolescents appear more vulnerable due to heightened sensitivity to social evaluation and less mature emotion-regulation capacities, as noted in adolescent sleep and cognitive studies by Scott and Woods and Short et al. [5,18]. Pre-existing mental health vulnerabilities further strengthen negative associations. Coyne et al. showed that adolescents with baseline depression or anxiety are more likely to engage in maladaptive use patterns, reinforcing bidirectional cycles of distress [22]. Content exposure also plays a critical moderating role, with repeated exposure to self-harm material, cyberbullying, or appearance-focused content markedly increasing psychological risk independent of overall time spent online [14,20,22]. Moderators influencing outcomes are shown in Table 1.

**Table 1:** Moderators Influencing the Impact of Social Media on Adolescent Mental Health

Moderator	Associated Outcomes	Source (Author, Year)
Gender	Stronger internalizing associations in girls	Twenge et al., 2019 [21]
Age	Greater vulnerability in early adolescence	Odgers et al., 2020 [1]; Rideout & Robb, 2021 [6]
Baseline mental health	Amplifies negative effects	Coyne et al., 2020 [22]
Type of use	Passive use worse; active use protective	Verduyn et al., 2017 [16]; Kross et al., 2021 [15]
Content exposure	Harmful content increases risk	John et al., 2018 [19]

Intervention Evidence

Intervention studies suggest that social media-related mental health risks are modifiable rather than inevitable. Experimental trials demonstrate that short-term reductions in social media use can lead to measurable improvements in psychological well-being, particularly among individuals with high baseline engagement. In a randomized controlled study, Hunt et al. showed that limiting social media use to approximately 30 minutes per day over three weeks resulted in significant reductions in loneliness and depressive symptoms compared with usual-use controls [23]. Similarly, Tromholt’s Facebook abstinence experiment found that participants who temporarily discontinued Facebook use reported higher subjective well-being, greater life satisfaction, and reduced negative affect, with the strongest effects observed among heavy users and those prone to passive consumption [24]. Together, these findings indicate that brief, low-intensity behavioral interventions targeting social media use can yield meaningful short-term mental health benefits, while also highlighting the need for adolescent-focused trials and longer follow-up to determine durability and developmental relevance. Sleep-focused interventions, including nighttime device restrictions, are supported by findings from Levenson et al. and Scott and Woods, which show that reducing bedtime use improves sleep and downstream mood outcomes [17,18]. At the family level, Livingstone and Helsper demonstrated that active parental mediation, characterized by discussion rather than restriction alone, is associated with healthier online behaviors and reduced risk exposure [25]. Family media plans involve collaboratively developed guidelines between caregivers and adolescents that outline expectations for social media use, including device-free periods (e.g., bedtime), content boundaries, and appropriate online behavior [11]. These plans emphasize communication and parental modeling rather than restriction alone and have been associated with lower rates of problematic use, improved sleep, and reduced exposure to online harms while preserving the social benefits of digital engagement [11,25]. School-based digital literacy programs aim to reduce psychiatric risk by targeting cognitive, emotional, and behavioral processes implicated in social media-related distress. These interventions incorporate psychoeducation on online stressors, training in emotion regulation and impulse control during digital interactions, and strategies to manage peer conflict and cyberbullying [26]. Programs such as iKeepSafe emphasize recognizing psychologically harmful content, responding adaptively to online harassment, and seeking support, which are clinically relevant skills for preventing anxiety, depressive symptoms, and stress-related dysregulation [27]. Reviews by Hutson et al. and Jones et al. indicate that such programs are associated with reductions in cyberbullying exposure and improvements in coping, emotional awareness, and help-seeking behaviors, although evidence for long-term psychiatric outcomes remains limited [26,27]. At the policy level, Montgomery et al. and Jia et al. argue that structural interventions targeting platform design and data practices are essential complements to individual-level strategies. Platform design features such as infinite scroll, algorithmic amplification, notification systems, and reward-based feedback are engineered to maximize engagement and can intensify compulsive use, social comparison, and emotional arousal. Data practices-including extensive data harvesting, personalized content targeting, and opaque recommendation algorithms-may disproportionately expose adolescents to emotionally salient or harmful content. Policy approaches emphasizing age-appropriate design, limits on data collection, algorithmic transparency, and safeguards against harmful content aim to reduce these risks while preserving opportunities for social connection and expression [28,29]. These intervention approaches are summarized in Table 2. 

**Table 2:** Intervention Approaches Addressing Social Media-Related Mental Health Risks

Level	Strategy	Reported Benefits	Supporting Sources (Author, Year)
Individual	Short-term breaks, mindfulness-based interventions	Improved mood, reduced loneliness	Hunt et al., 2018 [23]; Tromholt, 2016 [24]; Huberty et al., 2019 [20]
Family	Media plans, parent–child communication	Reduced problematic use, improved sleep	Livingstone & Helsper, 2008 [25]; George et al., 2018 [11]
School	Digital literacy and cyberbullying prevention programs	Increased resilience, reduced cyberbullying	Hutson et al., 2018 [26]; Jones et al., 2014 [27]
Policy	Platform design regulation, data protection	Structural risk reduction	Montgomery et al., 2017 [28]

Discussion

This narrative review highlights the complex and context-dependent relationship between social media use and adolescent mental health. Across studies published between 2007 and 2025, evidence consistently indicates that social media influences mental health through multiple, interacting pathways rather than a single causal mechanism [8,14,17]. Although average effect sizes linking social media use to depression and anxiety are modest, the cumulative impact is meaningful at the population level due to the near-universal exposure of adolescents during a developmentally sensitive period [5,17]. The findings of this narrative review are consistent with prior large-scale reviews indicating that associations between social media use and adolescent mental health outcomes are generally modest but reliable and highly context dependent [1,17]. Similar to earlier syntheses, overall screen time shows weak and inconsistent associations with depression and anxiety, whereas specific patterns of use, such as passive consumption, nighttime engagement, and exposure to harmful content, are more strongly linked to adverse outcomes [1,5,17]. This review extends previous work by integrating risk and protective pathways, mechanisms, moderators, and multilevel interventions within a single developmental framework, thereby emphasizing clinically relevant distinctions between types of engagement rather than duration alone [8,17].

A central finding across the literature is that how adolescents use social media matters more than how much they use it. Passive consumption, emotionally invested engagement, and problematic or compulsive use patterns are more reliably associated with internalizing symptoms than overall time spent online [5,13,14]. This distinction helps reconcile inconsistent findings in earlier research that relied primarily on screen-time metrics and underscores the importance of assessing qualitative aspects of digital engagement in both research and clinical settings [5,17]. Bidirectional effects further complicate interpretation, as adolescents experiencing emotional distress may increase social media use for distraction or connection, while maladaptive patterns of use can, in turn, exacerbate symptoms [17,22]. Several risk pathways appear particularly salient. Sleep disruption emerges as one of the most robust and biologically plausible mediators, with nighttime social media use contributing to delayed sleep onset, reduced sleep quality, and downstream effects on mood and emotional regulation [10-12]. Cyberbullying represents another high-impact pathway, consistently associated with elevated risk for depression, anxiety, self-harm, and suicidal ideation, with effect sizes exceeding those observed for general social media use [15,16]. Together, these findings suggest that content-specific and behavior-specific risk assessment may be more clinically informative than global recommendations regarding social media exposure [14,26].

Importantly, this review also demonstrates that social media can function as a source of resilience and support, particularly when engagement occurs within supportive social contexts and involves active, affirming interactions. Social connection, identity exploration, access to mental health information, and participation in affirming online communities are associated with improved well-being and reduced loneliness, especially among adolescents with limited offline support or marginalized identities [9,14,15,20]. These protective pathways highlight the limitations of abstinence-based approaches and support strategies that aim to promote healthy, intentional engagement rather than complete avoidance [9,17]. The heterogeneous impact of social media is further explained by moderating factors, including gender, age, pre-existing mental health vulnerability, type of use, and content exposure [5,14,17]. Younger adolescents and those with existing emotional difficulties appear more vulnerable to negative effects, while active, socially oriented use is often protective [5,14,20]. This variability reinforces the need for individualized, developmentally informed guidance rather than one-size-fits-all recommendations [17].

Intervention evidence suggests that social media-related risks are modifiable through coordinated, multilevel strategies. Individual-level approaches such as short-term use reduction, screen curfews, and mindfulness-based digital interventions show promising effects on mood and sleep [20,22-24]. Family-level strategies emphasizing communication and shared media practices, school-based digital literacy programs, and emerging policy-level reforms targeting platform design and accountability offer complementary avenues for risk reduction [25-28]. From a policy and public health perspective, increasing attention has been directed toward age-appropriate platform design, algorithmic transparency, and stronger protections against harmful content and cyberbullying as essential components of adolescent mental health prevention. Age-appropriate design frameworks emphasize default privacy settings, limits on persuasive design features (e.g., infinite scroll, streaks, and push notifications), and reduced exposure to social comparison cues for younger users, with the goal of minimizing excessive engagement and emotional dysregulation during a neurodevelopmentally sensitive period [28,29]. Algorithmic transparency represents a complementary priority, as opaque recommendation systems can amplify emotionally charged, appearance-focused, or distress-related content, potentially reinforcing maladaptive social comparison, anxiety, and compulsive use patterns. Greater transparency and accountability in content-ranking algorithms may allow for external oversight, improved risk mitigation, and intentional prioritization of developmentally appropriate or supportive content [29]. Finally, stronger safeguards against harmful content and cyberbullying-including proactive detection systems, reporting mechanisms, and consistent enforcement are critical given the robust associations between online harassment and depression, anxiety, self-harm, and suicidality in adolescents. Together, these structural interventions shift responsibility from individual users to platform-level design and governance, aligning digital environments more closely with adolescent developmental needs while preserving opportunities for connection and expression [28,29].

In sum, social media should not be conceptualized as inherently harmful to adolescent mental health. Rather, it represents a powerful social environment whose impact depends on patterns of use, individual vulnerability, and contextual supports [9,14,17]. Future research should prioritize longitudinal and experimental designs, improved measurement of engagement quality, and evaluation of platform-level interventions. Clinically and from a public health perspective, the goal should shift from limiting exposure to optimizing digital environments and supporting adolescents in developing healthy, adaptive patterns of use.

Strengths and Limitations

This review synthesizes nearly two decades of multidisciplinary evidence to provide a comprehensive, developmentally informed perspective on social media use and adolescent mental health. By integrating risk and protective pathways, underlying mechanisms, moderating factors, and intervention strategies, it offers a clinically and conceptually useful framework that extends beyond time-based measures of exposure. As a narrative review, the literature search was deliberately non-systematic to allow integration of diverse methodologies, disciplinary perspectives, and rapidly evolving evidence that would be difficult to capture within rigid systematic review criteria. While this approach may introduce selection bias, it is well suited to the review’s objective of synthesizing complex and heterogeneous findings across psychology, psychiatry, public health, and digital media research. The reviewed studies varied in design and measurement, and much of the evidence remains observational, limiting causal inference; additionally, ongoing changes in platform features may affect the relevance of older studies. These limitations underscore the need for continued longitudinal, experimental, and platform-level research rather than detracting from the value of the present synthesis.

## Conclusions

This narrative review demonstrates that social media’s impact on adolescent mental health is complex and context-dependent, shaped by patterns of use, individual vulnerability, and contextual factors rather than exposure alone. Even modest associations with depression and anxiety may have significant population-level impact due to the near-universal use of social media during adolescence. These findings support a shift away from time-based restrictions toward developmentally informed, multilevel strategies that promote healthy engagement and safer digital environments. From a policy perspective, age-appropriate platform design, algorithmic transparency, and stronger protections against harmful content and cyberbullying are essential to mitigating risk while preserving the developmental benefits of social media use. From a psychiatric perspective, routine clinical assessments should include targeted evaluation of social media use, with attention to content exposure, sleep disruption, social comparison, and cyberbullying experiences, particularly in adolescents presenting with mood or anxiety symptoms. Incorporating psychoeducation and collaborative digital-use guidance into treatment planning may help mitigate risk while supporting adaptive engagement.

## References

[1] Odgers CL, Schueller SM, Ito M (2020). Screen time, social media use, and adolescent development. Annual Rev Develop Psychol.

[2] Casey BJ, Jones RM, Hare TA (2008). The adolescent brain. Ann N Y Acad Sci.

[3] Merikangas KR, He JP, Burstein M (2010). Lifetime prevalence of mental disorders in U.S. adolescents: results from the National Comorbidity Survey Replication--Adolescent Supplement (NCS-A). J Am Acad Child Adolesc Psychiatry.

[4] Boyd DM, Ellison NB (2007). Social network sites: definition, history, and scholarship. Comput Mediat Commun.

[5] Short MA, Gradisar M, Gill J, Camfferman D (2013). Identifying adolescent sleep problems. PLoS One.

[6] Rideout V, Robb MB (2021). The Common Sense Census: Media Use by Tweens and Teens. https://www.commonsensemedia.org/sites/default/files/research/report/8-18-census-integrated-report-final-web_0.pdf.

[7] Odgers CL, Jensen MR (2020). Annual Research Review: adolescent mental health in the digital age: facts, fears, and future directions. J Child Psychol Psychiatry.

[8] Ophir E, Nass C, Wagner AD (2009). Cognitive control in media multitaskers. Proc Natl Acad Sci U S A.

[9] Uncapher MR, K Thieu M, Wagner AD (2016). Media multitasking and memory: differences in working memory and long-term memory. Psychon Bull Rev.

[10] Montag C, Lachmann B, Herrlich M, Zweig K (2019). Addictive features of social media/messenger platforms and freemium games against the background of psychological and economic theories. Int J Environ Res Public Health.

[11] George MJ, Russell MA, Piontak JR, Odgers CL (2018). Concurrent and subsequent associations between daily digital technology use and high-risk adolescents' mental health symptoms. Child Dev.

[12] Przybylski AK, Murayama K, DeHaan CR, Gladwell V (2013). Motivational, emotional, and behavioral correlates of fear of missing out. Comp Human Behavior.

[13] Vogel EA, Rose JP, Roberts LR, Eckles K (2014). Social comparison, social media, and self-esteem. Psychol Pop Media Cult.

[14] Baker GZ, Krieger H, LeRoy SA (2016). Fear of missing out: relationships with depression, mindfulness, and physical symptoms. Trans Issues Psychol Sci.

[15] Kross E, Verduyn P, Sheppes G, Costello CK, Jonides J, Ybarra O (2021). Social media and well-being: pitfalls, progress, and next steps. Trends Cogn Sci.

[16] Verduyn P, Ybarra O, Résibois M, Jonides J, Kross E (2017). Do social network sites enhance or undermine subjective well-being? A critical review. Soc Issues Policy Rev.

[17] Levenson JC, Shensa A, Sidani JE, Colditz JB, Primack BA (2017). Social media use before bed and sleep disturbance among young adults in the United States: a nationally representative Study. Sleep.

[18] Scott H, Woods HC (2018). Fear of missing out and sleep: cognitive behavioural factors in adolescents' nighttime social media use. J Adolesc.

[19] John A, Glendenning AC, Marchant A (2018). Self-harm, suicidal behaviours, and cyberbullying in children and young people: systematic review. J Med Internet Res.

[20] Huberty J, Green J, Glissmann C, Larkey L, Puzia M, Lee C (2019). Efficacy of the mindfulness meditation mobile app “Calm” to reduce stress among college students: randomized controlled trial. JMIR Mhealth Uhealth.

[21] Twenge JM, Martin GN, Spitzberg BH (2019). Trends in U.S. Adolescents’ media use, 1976-2016: the rise of digital media, the decline of TV, and the (near) demise of print.. Psychol Pop Media Cult.

[22] Coyne S, Rogers A, Zurcher J, Stockdale L, Booth M (2020). Does time spent using social media impact mental health?: an eight year longitudinal study. Comp Human Behavior.

[23] Hunt MG, Marx R, Lipson C, Young J (2018). No more FOMO: limiting social media decreases loneliness and depression. J Soc Clin Psychol.

[24] Tromholt M (2016). The Facebook experiment: quitting Facebook leads to higher levels of well-being. Cyberpsychol Behav Soc Netw.

[25] Livingstone S, Helsper EJ (2008). Parental mediation of children’s internet use. J Broadcast Electronic Media.

[26] Hutson E, Kelly S, Militello LK (2018). Systematic review of cyberbullying interventions for youth and parents with implications for evidence‐based practice. Worldviews Evid Based Nurs.

[27] Jones M, Mitchell J, Walsh A (2014). A content analysis of youth internet safety programs: are effective prevention strategies being used?. University of New Hampshire Scholars' Repository.

[28] Montgomery C, Chester J, Milosevic T (2017). Ensuring young people’s digital privacy as a fundamental right. International Handbook of Media Literacy Education.

[29] Jia L, Nieborg B, Poell T (2022). On super apps and app stores: digital media logics in China’s app economy. Media Culture Soc.

